# Acute Pancreatitis Caused by Pegylated (PEG)-Asparaginase Associated With Severe Hypertriglyceridemia

**DOI:** 10.7759/cureus.62448

**Published:** 2024-06-15

**Authors:** Sumina Rai, Prabhat Sharma, Anamika Nepal, Sajana Poudel, Yannis Guerra

**Affiliations:** 1 Internal Medicine, John H. Stroger, Jr. Hospital of Cook County, Chicago, USA; 2 Internal Medicine, Shankarapur Hospital, Kathmandu, NPL; 3 Endocrinology, Diabetes and Metabolism, John H. Stroger, Jr. Hospital of Cook County, Chicago, USA

**Keywords:** leukemia, acute lymphoblastic leukemia (all), chemotherapy agents, chemotherapy-related toxicity, b-all, hypertriglyceridemia, peg-asparaginase, acute pancreatitis

## Abstract

Pegylated (PEG)-asparaginase is used during the induction and intensification phases of treatment for B-cell acute lymphoblastic leukemia (B-ALL). It works by depleting the external sources of asparagine, causing the death of lymphoblasts. It has several adverse effects, including pancreatitis and hypertriglyceridemia; however, the simultaneous occurrence of both is uncommon. We present the case of an 18-year-old man with B-ALL who developed acute epigastric pain radiating to the back and non-bloody, non-bilious emesis following treatment with PEG-asparaginase. He was diagnosed with acute interstitial pancreatitis and severe hypertriglyceridemia. Conservative management was used for the pancreatitis, while hypertriglyceridemia was treated with an insulin infusion. Pancreatic toxicity and hypertriglyceridemia can necessitate the discontinuation of PEG-asparaginase, limiting treatment options and potentially increasing the risk of relapse. Therefore, further studies are needed to identify the factors contributing to hypertriglyceridemia and pancreatitis, aiding clinicians in monitoring and prevention.

## Introduction

The key component of treating B-cell acute lymphoblastic leukemia (B-ALL) is pegylated (PEG)-asparaginase with glucocorticoids and other chemotherapeutic agents [1]. Asparaginase is used in B-ALL during the induction and intensification phases of treatment [2]. The primary target of asparaginase is malignant lymphoblasts, which work by causing the depletion of the external sources of asparagine through the hydrolysis of asparagine into aspartic acid [3]. As most malignant lymphoblasts have limited asparagine synthetase activity to synthesize asparagine, its lack leads to apoptosis of the lymphoblasts [4]. There are three formulations of asparaginase available: L-asparaginase, PEG-asparaginase, and Erwinia asparaginase [4]. Asparaginase has been used for over 50 years. Among the different formulations, PEG-asparaginase has the benefit of having a long half-life of six days and fewer adverse effects than others [5]. There are several adverse effects of PEG-asparaginase, which are pancreatitis, hypertriglyceridemia, hepatic injury, thrombosis, hypersensitivity reactions, and hyperviscosity syndrome [5]. We present the case of a patient with B-ALL who was treated with PEG-asparaginase, which was complicated by acute pancreatitis and severe hypertriglyceridemia.

## Case presentation

Our patient was an 18-year-old gentleman with high-risk B-ALL undergoing treatment. He presented to the ED with a one-day history of acute, severe, constant epigastric pain radiating to the back, which worsened with eating. He also experienced nausea and had two episodes of non-bloody, non-bilious emesis. He denied fever, chills, chest pain, shortness of breath, dysuria, diarrhea, constipation, alcohol intake, cannabinoid use, and herbal supplementation. There was no prior history of similar episodes or any personal or family history of hypertriglyceridemia.

For B-ALL, the patient was enrolled in the Children’s Oncology Group’s AALL1732 Phase 3 randomized trial, which evaluates inotuzumab ozogamicin for newly diagnosed patients with high-risk B-ALL. He achieved minimal residual disease negative remission at the end of induction. He then received intensified chemotherapy with PEG-asparaginase and dexamethasone 18 days before the onset of symptoms. Additionally, seven days before admission, he underwent delayed intensification chemotherapy with intravenous vincristine and doxorubicin and started a seven-day course of dexamethasone.

Upon arrival, the patient was afebrile with a heart rate of 105 beats per minute, a respiratory rate of 22 breaths per minute, a blood pressure of 126/83 mm Hg, and an oxygen saturation of 97% on room air. His BMI was 22 kg/m². He appeared to be in mild distress with dry mucous membranes. Abdominal examination revealed moderate epigastric tenderness with voluntary guarding. Relevant laboratory results on presentation are summarized in Table 1.

**Table 1:** Laboratory results on presentation VBG, venous blood gas

Laboratory results	Value	Reference range
WBC count	16,000	4,000-11,000 cells/mm^3^
Total neutrophils	15,040	2,500-8,000 cells/mm^3^
Total lymphocytes	384	1,000-4,000 cells/mm^3^
Total blastocytes	0	100-700 cells/mm^3^
Hemoglobin	15	12.0-16.0 g/dL
Platelet count	386,000	150,000-400,000 cells/mm^3^
Sodium	128	135-148 mmol/L
Potassium	4.4	3.5-5.3 mmol/L
Chloride	88	98-110 mmol/L
Bicarbonate	20	21.0-32.0 mmol/L
Blood urea nitrogen	25	7-20 mg/dL
Creatinine	0.8	0.6-1.0 mg/dL
Calcium	8.8	8.8-10.5 mg/dL
Lipase	954	0-160 U/L
Triglycerides	2042	<150 mg/dL
Alanine aminotransferase	58	20-65 U/L
Aspartate aminotransferase	26	15-37 U/L
Total bilirubin	1.3	0.0-1.0 mg/dL
Direct bilirubin	0.2	<0.3 mg/dL
Alkaline phosphatase	246	50-136 U/L
Gamma-glutamyl transferase	125	5-750 IU/L
Random blood glucose	170	80-140 (mg/dl)
VBG pH	7.24	7.35-7.45
VBG CO_2_	44.5	35-45 mm Hg
VBG bicarbonate	19.5	22-26 mEq/L
VBG lactate	4.95	<2 mmol/L

**Figure 1:**
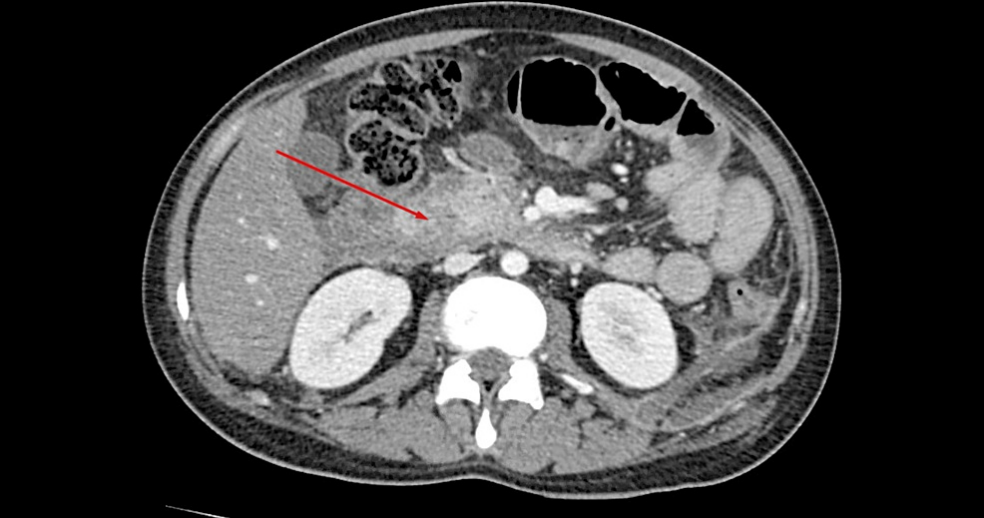
Contrast-enhanced CT of the abdomen showing features consistent with acute pancreatitis, including peripancreatic fluid collection (indicated by the red arrow)

The chest X-ray was normal. An initial CT scan of the abdomen and pelvis revealed acute interstitial edematous pancreatitis with peripancreatic fluid collection (Figure 1). A gallbladder ultrasound showed no biliary dilatation or gallstones.

The patient was admitted to the medical intensive care unit, where he received intravenous fluids, antibiotics, and supportive treatment. The non-diabetic ketoacidosis protocol was initiated with insulin at 1 unit/hour to treat hypertriglyceridemia. His hospital course was complicated by acute hypoxic respiratory failure due to bilateral pleural effusions and paralytic ileus, both of which were managed conservatively. A repeat CT scan of the abdomen revealed necrotizing pancreatitis, which required no surgical intervention as there were no signs of infection. He gradually improved, and his triglyceride (TG) levels began to decrease. When TG levels dropped below 1,000 mg/dL, he was transitioned off the insulin drip to fenofibrate. By the ninth day of admission, his TG levels had decreased to 151 mg/dL. He was discharged home after a 10-day hospital stay. Relevant laboratory results on the day of discharge are summarized in Table 2.

**Table 2:** Laboratory results on the day of discharge

Laboratory results	Value	Reference range
WBC count	4,000	4,000-11,000 cells/mm^3^
Total neutrophils	2,800	2,500-8,000 cells/mm^3^
Total lymphocytes	700	1,000-4,000 cells/mm^3^
Total blastocytes	0	100-700 cells/mm^3^
Hemoglobin	8.6	12.0-16.0 g/dL
Platelet count	359,000	150,000-400,000 cells/mm^3^
Sodium	141	135-148 mmol/L
Potassium	3.8	3.5-5.3 mmol/L
Chloride	104	98-110 mmol/L
Bicarbonate	30	21.0-32.0 mmol/L
Blood urea nitrogen	4	7-20 mg/dL
Creatinine	0.4	0.6-1.0 mg/dL
Calcium	7.8	8.8-10.5 mg/dL
Lipase	350	0-160 U/L
Triglycerides	133	<150 mg/dL
Alanine aminotransferase	34	20-65 U/L
Aspartate aminotransferase	28	15-37 U/L
Total bilirubin	0.6	0.0-1.0 mg/dL
Direct bilirubin	0.2	<0.3 mg/dL
Alkaline phosphatase	212	50-136 U/L
Gamma-glutamyl transferase	95	5-750 IU/L
Random blood glucose	143	80-140 (mg/dl)

**Figure 2:**
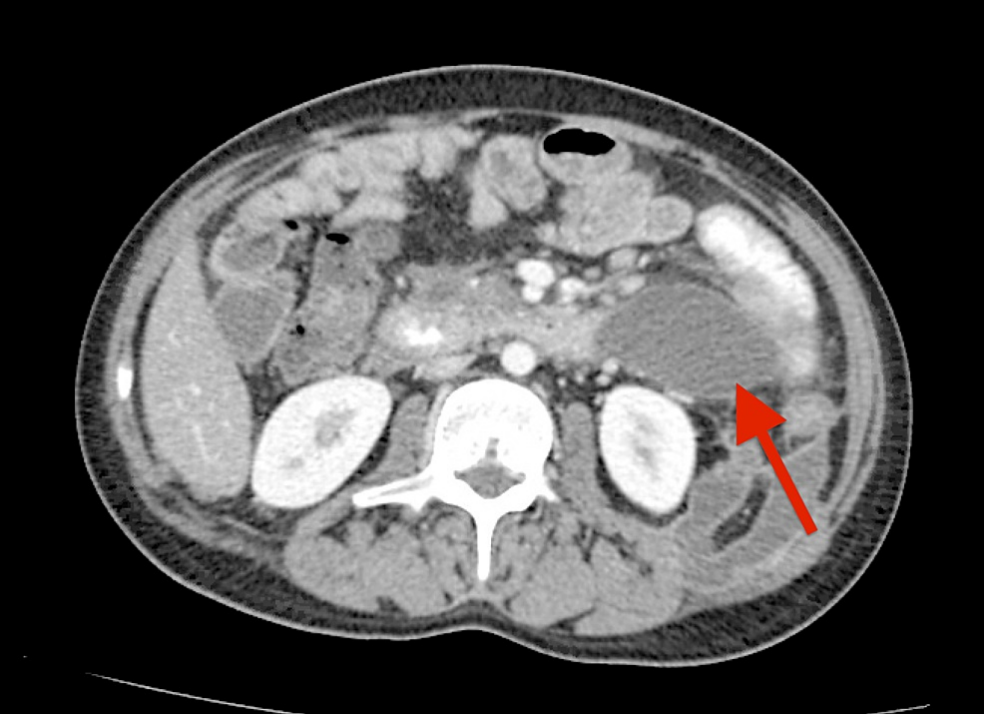
CT abdomen showing healing necrotizing pancreatitis complicated by the formation of a multiloculated pseudocyst (indicated by the red arrow)

Fifteen days later, the patient presented with mild abdominal pain. Lab workup revealed a lipase level of 300 U/L and TG of 106 mg/dL, and a CT scan of the abdomen and pelvis showed necrotizing pancreatitis with complex-appearing ascites, including loculated fluid. He was managed conservatively, showed clinical improvement, and was discharged. At the post-hospital visit two weeks later, the patient’s TG level was 96 mg/dL. A repeat CT scan revealed healing necrotizing pancreatitis complicated by the formation of a multiloculated pseudocyst (Figure 2). He was planned for future endoscopic stenting. Chemotherapy was kept on hold, with the plan to resume after stenting. Unfortunately, the patient was lost to follow-up in our center.

## Discussion

In isolation, asparaginase can be the primary culprit behind acute pancreatitis. Given its association with hypertriglyceridemia, the onset of acute pancreatitis can also be attributed to it [1,2,4]. The origin, formulation, dosage, or method of administration of asparaginase do not determine the risk of pancreatitis [4]. The exact pathophysiology of acute pancreatitis with asparaginase is unknown. Still, it could be related to asparagine depletion in organs with high protein turnover, such as the liver or pancreas [4].

The combination treatment of glucocorticoids and asparaginase can alter the lipid profile, particularly in hypertriglyceridemia [1]. Among the different preparations of asparaginase, the risk of hypertriglyceridemia has been observed to be increased with PEG-asparaginase compared to L-asparaginase almost two times when given together with dexamethasone [6]. Most episodes of hypertriglyceridemia reported have been within two weeks of the administration of PEG-asparaginase and steroids. PEG-asparaginase causes elevated TG levels by inhibiting lipoprotein activity, resulting in elevated exogenous chylomicrons and increased endogenous very low-density lipoprotein production [5]. Glucocorticoids increase the synthesis of TGs and mobilize fatty acids while activating the lipoprotein lipase activity responsible for the hydrolysis of triglycerides [1].

In patients with ALL treated with asparaginase, the association between severe hypertriglyceridemia and pancreatitis is rarely seen [6]. Two patients who developed hypertriglyceridemia >4,000 mg/dl after treatment with PEG-asparaginase did not develop acute pancreatitis or skin manifestations of hypertriglyceridemia [5]. Singh et al. reported hypertriglyceridemia >13,000 mg/dl with L-asparaginase without evidence of pancreatitis, which was treated with intravenous insulin infusion and low-intensity atorvastatin, resulting in the normalization of the lipid profile [7]. Based on these observations, the most likely trigger for pancreatitis in our patient was PEG-asparaginase rather than hypertriglyceridemia.

The factors making patients more susceptible to these adverse effects are unclear. Genetic predisposition can play a role in determining which patients are more vulnerable to pancreatitis [4]. In the study by Finch et al., genetic predisposition to hypertriglyceridemia was not significant in patients treated with PEG-asparaginase [6]. A recent study reveals that individuals harboring a rare heterozygous missense variant c.11G > A-p (Arg4Gln) within the APOC3 gene, categorized as a variant of uncertain significance, exhibited elevated plasma TG levels. This association was linked to four common single nucleotide polymorphisms [1].

The principle of treatment for acute pancreatitis secondary to PEG-asparaginase is similar to the treatment of acute pancreatitis due to any other cause [4]. Early fluid resuscitation and the early start of enteral nutrition are important [4]. For treating severe hyperglyceridemia, various insulin regimens, including continuous and intermittent infusions, are suggested [8]. For non-diabetic patients, additional considerations should be made for the prevention of hypoglycemia [8]. Our patient was treated with an insulin infusion for hypertriglyceridemia.

These adverse effects of asparaginase often lead to its discontinuation during treatment [1]. However, some studies have been done in which asparaginase was reintroduced in select patient populations with mild acute pancreatitis who had complete resolution of symptoms. Three studies reported recurrence of pancreatitis in 0%, 7.7%, and 62.5% of the patients included in their studies, respectively [4]. Failure to complete the planned asparaginase treatment has been associated with an increased relapse of the disease [4].

## Conclusions

PEG-asparaginase is an integral part of the chemotherapy regimen for B-ALL. Pancreatic toxicity is a significant possible adverse effect of PEG-asparaginase, the occurrence of which can lead to its discontinuation. It is crucial to balance, on a case-by-case basis, the risk of recurrence of pancreatitis versus the potential benefit of reintroducing the chemotherapeutic agent. Further studies need to be done to identify the factors that play a role in causing hypertriglyceridemia and pancreatitis. It would help to prevent such serious complications and ensure continued chemotherapy. Also, the role of lipid-lowering therapy needs to be studied to prevent or reduce the severity of hypertriglyceridemia.
